# In-House Validation of an SPE-GC-FID Method for the Detection of Free and Esterified Hydroxylated Minor Compounds in Virgin Olive Oils

**DOI:** 10.3390/foods10061260

**Published:** 2021-06-02

**Authors:** Enrico Valli, Andrea Milani, Ana Srbinovska, Erica Moret, Sabrina Moret, Alessandra Bendini, Wenceslao Moreda, Tullia Gallina Toschi, Paolo Lucci

**Affiliations:** 1 Department of Agricultural and Food Sciences, Alma Mater Studiorum—Università di Bologna, 40127 Bologna, Italy; enrico.valli4@unibo.it (E.V.); alessandra.bendini@unibo.it (A.B.); tullia.gallinatoschi@unibo.it (T.G.T.); 2 Department of Agri-Food, Animal and Environmental Sciences, University of Udine, via Sondrio 2/a, 33100 Udine, Italy; milani.andrea.1@spes.uniud.it (A.M.); srbinovska.ana@spes.uniud.it (A.S.); erica.moret@uniud.it (E.M.); sabrina.moret@uniud.it (S.M.); 3 Department of Characterization and Quality of Lipids, Instituto de la Grasa-CSIC, Campus of Universidad Pablo de Olavide, E-41013 Sevilla, Spain; wmoreda@ig.csic.es

**Keywords:** olive oil, authenticity, sterols, hydroxylated minor compounds, purity, quality, fraud, esterified compounds, in-house validation

## Abstract

Minor compounds in vegetable oils are distributed between free and esterified forms, and the ratio of these two fractions could represent an important parameter for assessment of oil authenticity. A simple method based on offline SPE-GC-FID for the analysis of free and esterified hydroxylated minor compounds in olive and sunflower oils has been developed and in-house validated. A satisfactory repeatability relative standard deviation (<7.5%) was obtained in all cases. The method, which requires simple instrumentation, allows for reliable quantification in a single chromatographic run with the advantages of minimizing sample manipulation, use of toxic solvents and reagents, and time consumption. The analytical procedure was applied to pure oil samples, including 15 authentic extra virgin olive oils collected from different European countries (Spain, Italy, Greece, and Portugal). Finally, the proposed SPE-GC-FID methodology could detect changes in the ratio between the free and esterified forms in pure extra virgin olive oil when mixed with refined sunflower oil at different percentages of 2, 5, 10, 15, and 20% (*w*/*w*) to simulate adulteration.

## 1. Introduction

The global trade of extra virgin olive oil (EVOO) has extensively increased during recent years due to its peculiar sensory characteristics, high nutritional values, and healthy benefits associated with its consumption [1,2]. As a result, EVOO and virgin olive oil (VOO), which are recognized as typical ingredients of the Mediterranean diet, are costly products and, therefore, fraud is frequent [3,4]. According to the Food Fraud Network Activity Report, in 2019 the category ‘fats and oils’ became the first in terms of number of requests created in the Administrative Assistance and Cooperation system placing ‘olive oil’ (OO) as the most notified product [5]. Particular attention must be thus paid to EVOO production and commercialization to guarantee compliance with quality and purity parameters specified by the EU, the International Olive Council (COI), and the Codex Alimentarius [6,7,8].

With respect to the analytical methods available for purity assessment of olive oil, evaluation of the composition of sterols is a well-established tool, as the sterol profile depends on the botanical origin of oils [9]. For instance, olive oil is characterized by high amounts of β-sitosterol and Δ5-avenasterol, together with low concentrations of campesterol, stigmasterol, and Δ5-stigmasterol. On the other hand, campesterol and stigmasterol are markers for the addition of sunflower, soybean, safflower, peanut, cottonseed, and palm oils, among others, to olive oil. Even if not thoroughly studied, other hydroxyl-bearing molecules, such as methyl sterols and triterpenic alcohols, are also characteristic of selected botanical origin [10,11]. In fact, in the last decades, the genetic improvement of edible seed oils has deeply modified their fatty-acid composition, making this analysis insufficient to highlight olive oil adulterations. The combination of chromatographic and spectroscopic methods (i.e., UV, NIR, MIR, etc.) together with advanced chemometric tools (principal component analysis (PCA), soft independent modelling of class analogy (SIMCA), partial least squares regression (PLSr), etc.) has been also exploited for the assessment of authenticity, as well as of the quality and geographical origin, of olive oils [12,13,14]. For instance, a recent study showed the usefulness of a PLS regression model for the characterization and classification of edible oils (olive, sunflower, soy, and corn oils) and olive oils from different olive cultivars (Arbequina, Picual, Hojiblanca, and Cornicabra) using polyphenolic fingerprints obtained by HPLC-DAD analysis [15]. Previous studies have also focused on the study of the volatile composition of extra virgin olive oils for the identification of discriminant compounds able to signify adulteration [16,17]. Similarly, Mohamed et al. [18] differentiated Tunisian and Italian extra virgin olive oils according to their phenolic and sterolic fingerprints by means of multivariate statistics. However, beside their great potentialities, the Achilles’ heel of untargeted and/or multitargeted analytical methodologies based on multivariate analysis is still represented by the absence of harmonized guidelines for full validation trials, which has to date prevented their adoption as official methods. Regarding official analytical approaches, the methods that currently are available for sterol analysis are suitable to determine the total composition of sterols [19,20,21]. The sample preparation provides a saponification step with KOH solution in methanol, followed by unsaponifiable liquid–liquid extraction (LLE) with diethyl ether, and a fractionation step by thin-layer chromatography (TLC), with subsequent derivatization of sterols for obtaining trimethylsilyl (TMS) derivatives to be analyzed by GC-FID. In this way, sterol concentration and relative amounts are obtained and compared to the values reported in the International Olive Council trade standard (IOC, 2019), Reg. (EEC) 2568/91, and following amendments and updating to detect adulterations or check the genuineness of the olive oil [8]. As mentioned, however, the official EU method for total sterol analysis is only suitable to determine the whole composition of sterols, and is not dependent on being in the free or in the esterified form. This latter aspect is particularly important when assessing the purity of an olive oil. In fact, a number of papers have now highlighted that minor compounds, including sterols, in plant and vegetable oils from both seeds and fruits may be in a free or esterified form, and that the ratio between these two forms is not constant and varies for different oils [22,23,24]. For this reason, the different distribution between free and esterified hydroxylated minor compounds (HMCs) could represent valuable information for distinguishing oils that present slight differences in whole sterol composition and to highlight the presence of illegal addition of extraneous oils (i.e., seed oils) in olive oil. In addition, the investigation of the profile of free and esterified minor compounds could also be useful to evaluate the degree of refining applied to an oil sample because of the different impact of the procedure on the distribution of free and esterified phytosterols that leads to a considerable increase in the steryl ester fraction [25]. There is, therefore, a need for a simple and reliable analytical method that is able to provide information about free and esterified HMCs that might be useful to discriminate between pure EVOO and fraudulent admixtures with refined seed oils.

Few attempts have been made to develop chromatographic methods that are able to characterize steryl ester and free fractions in olive oil. To date, there is no validated method that can be used for assessment of free and esterified HMCs in EVOO. In addition to this crucial aspect, previously published methods also had critical analytical aspects such as the requirement for saponification of the purified free and esterified fractions [24,25,26], a long preparative glass column employing significant amount of silica and solvents, and complex chromatographic approaches such as online LC × GC, demanding dedicated instrumentation and skilled operators [22,27,28,29,30,31].

Herein, a simplified method based on offline SPE-GC-FID for analysis of free and esterified HMCs (free and esterified sterols, free and esterified triterpenic alcohols), in olive and seed oils has been developed and in-house validated. The method has the advantage of being accessible to most analytical laboratories, since no expensive dedicated instruments are required. In addition, particular attention has been devoted to replacing the toxic solvents that are usually employed in sterol analysis, as well as to reducing the amounts of solvents and reagents needed for the procedure.

The proposed method has been used to characterize pure oil samples, including 15 authentic EVOOs collected from different European countries (Spain, Italy, Greece, and Portugal) within the EU Horizon 2020 OLEUM project.

Finally, the proposed SPE-GC-FID methodology has been applied to the determination of free and esterified HMCs, as well as the ratio between the two forms in pure EVOOs mixed in different proportions, to simulate EVOO adulteration with refined sunflower oil at different percentages of 2, 5, 10, 15, and 20% (*w*/*w*).

## 2. Materials and Methods

### 2.1. Chemicals and Reagents

All chemicals and reagents were obtained from Sigma-Aldrich Chemical Co., Ltd. (Milan, Italy). The isooctane (2,2,5 tri-methyl-pentane), *n*-heptane, *n*-hexane, and diethyl ether were of analytical grade. Internal standards α-cholestanol and cholesteryl palmitate were purchased from Sigma-Aldrich (Milan, Italy). Internal standards were prepared weekly using *n*-heptane as the diluting solvent. All solutions were stored at 4 °C. Silica gel (pore size 60 Å, 0.04–0.015 µm particle size) was purchased from Agilent (Milan, Italy).

### 2.2. Oil Samples

Pure EVOOs used for the in-house validation of the analytical method were obtained within the frame of the EU Horizon 2020 OLEUM Project (grant agreement ID: 635690). For this purpose, a set of 15 commercial authentic virgin olive oils produced during the olive oil seasons 2018–2019 were collected directly from olive oil mills or olive oil retailers. These samples were produced in different European countries: 8 in Greece, 3 in Spain, 2 in Portugal, and 2 in Italy. Other oil samples analyzed, such as refined sunflower oil (RSO), refined high-oleic sunflower oil (HOSO), and refined pomace olive oil (PO), were purchased at local markets. The samples were stored in the dark at −18 °C until chemical analyses were conducted.

### 2.3. Sample Preparation

For oil sample preparation, 150 µL of α-cholestanol at 0.1 mg/mL in *n*-heptane (to be used as internal standard for quantitative analysis of free minor compounds) and of cholesteryl palmitate at 0.1 mg/mL in *n*-heptane (to be used as internal standard for quantitative analysis of esterified minor compounds) were transferred to a test tube. The solvent was evaporated under a gentle nitrogen stream at room temperature, and 50 mg (±1 mg) of oil sample was carefully weighed in the glass tube. The sample was combined with 100 µL of BSTFA-TMCS (99:1, *v*/*v*) and 100 µL of pyridine, the tube was closed, the sample was mixed, and the reaction was carried out at room temperature for 20 min, after which the reaction mixture was evaporated by means of a soft nitrogen stream, and 0.5 mL of isooctane was added.

### 2.4. Extraction and Clean-Up Using Silica SPE

Empty 6 mL glass SPE cartridges were packed, transferring 1 g of silica between two frits. The silica packing bed was adjusted by pressing the upper frit with the superior part of a glass Pasteur pipette or a small lab spatula. Packing was carried out under dry conditions without the aid of solvent. Regarding the SPE protocol, 5 mL of isooctane was used for cartridge conditioning. Next, the sample solution, prepared as described in Section 2.3, was loaded into the SPE cartridge and the solvent eluted just above the frit on top of the silica; this volume of solvent was discarded. Minor compounds were then eluted with 20 mL of fresh isooctane/diethyl ether 99/1 (*v*/*v*) mixture and collected in a conical balloon. During this step, the column was not allowed to run dry. Vacuum was applied to obtain a flow of about 1 drop every second. The recovered solution was then evaporated to dryness by a vacuum rotary evaporator. Finally, the residue was dissolved in 0.2 mL of *n*-heptane for GC analysis carried out using a cold on-column injection port.

### 2.5. GC-FID Analysis

The GC analysis of minor compounds was carried out in a Carlo Erba Mega 5300 Gas chromatograph equipped with a cold on-column injector and a flame ionization detector (FID) (Fison’s MEGA 5300—Fison’s, Milan, Italy). A Mega 5 column (10 m × 0.32 mm × 0.1 µm) was used. The following operative conditions were applied: the temperature of detector was set at 350 °C, the temperature of the oven was programmed at 80 °C (1 min isotherm), then increased to 200 °C at a rate of 20 °C/min, then to 220 °C at a rate of 2 °C/min (to obtain a better separation between the free sterols’ internal standard and α-tocopherol, otherwise there would be a coelution of the two analytes), and lastly to 340 °C at a rate of 5 °C/min. The final isotherm was maintained for 15 min. Helium was used as a carrier gas at a constant pressure of 30 kPa, with hydrogen at 50 kPa and air at 80 kPa. The injection volume was 1 µL. Peaks were tentatively identified by comparison of relative retention times of authentic reference standards and elution profiles reported elsewhere [32]. Relative retention times of individual free and esterified HMCs are included in Supplementary Table S1.

## 3. Results and Discussion

### 3.1. SPE Method Optimization

Only a few investigations have dealt with determination of both free and esterified minor compounds in olive oil. The analytical methodologies described so far isolated esterified and free sterols mainly by either silica glass column or TLC, followed by saponification and GC analysis of both fractions [10,33,34,35]. In general, the polarity difference between the free compounds’ polar fraction and the esterified compounds’ apolar fraction is exploited in order to separate these two forms. However, the separation is usually conducted using a glass chromatographic column packed with large amount of silica (15–25 g), thus requiring large volumes of solvents. For instance, Verleyen et al. (2002), published a method using 75 mL of *n*-hexane/ethyl acetate (90:10% *v*/*v*) followed by elution with 75 mL *n*-hexane/diethyl ether/ethanol (25:25:50% *v*/*v*) for the isolation in edible oils of steryl ester and free sterol fractions, respectively [24]. Similarly, Mariani et al. (2006) developed a methodology based on a preparative chromatographic column packed with 25 g of silica gel in 80 mL of *n*-hexane and requiring 150 mL of a mixture of *n*-hexane/diethyl ether (87:13 *v*/*v*) to separate the esterified fraction [30]. Next, 150 mL of diethyl ether was employed for the polar fraction elution. At the end, more than 350 mL of solvent was required to conduct the analytical procedure. However, even when SPE was used as a reducing reagent and solvent consumption was compared to longer glass funnel, independent saponifications of free and esterified fractions were usually reported [33,35]. This means that a double GC run was required, which inevitably leads to more time-consuming procedures.

In light of this, for the optimization of the analytical procedure proposed herein for purification of HMCs, SPE was considered because, in addition to the above-mentioned advantages, more homogeneous packing of the silica bed can be obtained compared to a glass column. This aspect could undoubtedly represent one of the main factors affecting the repeatability of the analyses. Therefore, the first step was direct derivatization of the EVOO sample with BSTFA + 1%TMCS reagent diluted in pyridine (1:1, *v*/*v*). This decreased the polarity of free HMCs, making them much closer in terms of polarity to the esterified ones and allowing both fractions to be eluted together in the next SPE purification step. Initial experiments were done using the purification procedure (with 70 mL of solvent for silica conditioning, as well as elution of *n*-alkanes and minor compounds) as proposed by Mariani et al. [36,37,38], but using a plastic SPE cartridge packed with 5 g silica instead of a 40 cm glass column with 3 g of silica. As a result, the adoption of 5 g SPE led to better repeatability (8 vs. 17%, as RSDr values for SPE and the glass column, respectively), probably because the 40 cm glass column silica-packing was more subject to variability, which may have led to higher data dispersion. The next step was to try to reduce the amount of silica to scale down the protocol, changing the 5 g silica SPE procedure to a 1 g silica SPE method and using proportionately lower amounts of materials, reagents, and solvents [39]. For this purpose and considering the thinner chromatographic bed obtained when using a lower amount of stationary phase, the use of a different type of silica with a smaller diameter of the particles (0.040–0.015 µm) was selected to improve the separation ability of the fractions of interest from triacylglycerols (TAGs). In fact, one of the most critical aspects in the optimization of the protocol is to elute both derivatized free and esterified HMCs while keeping all TAGs in the SPE column, as these might otherwise determine important GC separation issues. At the same time, since there is a growing pressure on reducing or even eliminating usage of toxic solvents in the laboratory, a further improvement of the methodology was made by substituting *n*-hexane employed in the SPE protocol with less toxic isooctane. However, an analytical problem was the release of monomers and oligomers from plastic SPE cartridges observed when employing isooctane. Such interferences eluted in the first part of the GC trace, thus potentially affecting the quantification of the analytes, especially for those eluting in the free compounds region. To solve this problem, the use of a glass SPE cartridge was considered necessary to obtain reliable analytical results. This improvement avoids characteristic “hills” of monomers and oligomers in the first part of the chromatogram that caused an overestimation of some peak areas of free minor compounds.

As a result, after silica conditioning with a reduced volume (5 mL) of isooctane and the addition of the sample, the elution of minor compounds was performed with only 20 mL of isooctane/ethyl ether 99:1 (% *v*/*v*) solution. Comparable results to the 5 g silica cartridge previously employed were obtained. In total, with the procedure employing 1 g silica SPE, only 25 mL were required, thus saving up to 45 mL of solvent.

Finally, the extract thus obtained and containing derivatized free and esterified HMCs was evaporated to dryness, dissolved in 0.2 mL of *n*-heptane, and then injected in GC by using a cold on-column injection port. The use of such injection mode is essential to prevent thermal degradation of esterified compounds, as well as to avoid any discrimination of high-boiling-point minor compounds during injection. In the meantime, the use of *n*-heptane as injection solvent permitted an initial temperature not too low (b.p. *n*-heptane is about 98 °C at 1 bar, which means the injection temperature could be about 65 °C), so that the resident time of the sample in the columns was quite acceptable.

**Figure 1:**
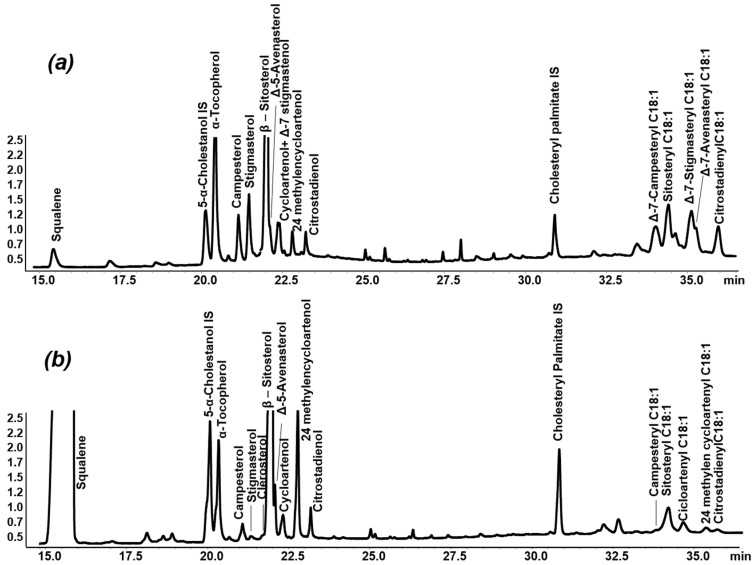
GC-FID chromatograms of TMS free and esterified minor compounds in refined high-oleic sunflower (**a**), and extra virgin olive (**b**) oils; for fatty acids. Note: m:n—m = number of carbon atoms, n = number of double bonds.

Chromatograms of TMS free and esterified compounds obtained by applying the optimized SPE-GC-FID procedure (1 g silica gel glass SPE column) are presented in Figure 1. As can be seen in the figure, EVOO and HOSO traces differed both quantitatively and qualitatively. Interestingly, in addition to the large and notorious differences in free minor compounds, the esterified fraction also revealed peculiar characteristics of the two selected oil samples. In fact, some esterified minor compounds, such as Δ-7-stigmasteryl C18:1 and Δ-7-avenasteryl C18:1, were not present in EVOO, whereas solely EVOO contained 24 methylen cycloartenyl C18:1. However, considering the objective of our study, the most interesting data was undoubtably represented by the higher content of esterified components in sunflower oil, which could be extremely useful for detecting its fraudulent addition to olive oil.

### 3.2. In-House Validation of the SPE-GC-FID Procedure

Once the method was optimized, the next step was “in-house” validation of the method using three different oils to check for, among other analytical quality parameters, the presence of possible matrix effects. For this purpose, EVOO, PO, and RSO samples were selected and submitted to the SPE-GC-FID procedure for quantification of total free and esterified HMC. The choice of these types of oils was made considering that they have different distribution of hydroxylated compounds between free and esterified fractions. EVOO is characterized by a lower concentration of esterified sterols if compared to PO, while RSO contains higher levels of Δ-7-stigmastenol, which is characteristic of Compositae oils (i.e., sunflower and safflower) and is mainly present in the esterified form. This aspect could be of particular importance, because if sterols are removed from RSO with the aim to obtain a high oleic sunflower oil without hydroxylated compound markers, free and esterified minor compounds are differently removed, with the latter being less deleted. To assess the repeatability of the methodology, each analysis was repeated 6 times. The results expressed as average content (mg/kg) of free and esterified minor compounds ± repeatability standard deviation and repeatability relative standard deviation relative (RSDr (%)) are reported in Table 1.

The results showed clear differences in terms of free and esterified contents of the three selected oil samples. As noted before for HOSO, RSO also contained higher levels of esterified minor compounds compared to both EVOO and PO. In particular, RSO showed Δ-7-stigmasteryl C18 content of 850 mg/kg, whereas this esterified sterol was absent in both EVOO and PO, suggesting its possible use as an esterified marker for RSO addition. However, it should be noted that PO also is characterized by a higher level of esterified components than EVOO. In general, and focusing on the repeatability of the methodology, slightly better results were obtained for the quantitative analysis of free minor compounds in RSO (4.2%) rather than EVOO and PO (5.9%). On the other hand, regarding measurements of esterified fractions, EVOO showed a lower RSDr value (4.6%) compared to the other oil samples. This result could be partially ascribed to the higher level of waxes in PO and RSO, which presented similar but higher RSD% (7.2 and 7.5% for PO and RSO, respectively) compared to EVOO.

In general, adequate results were therefore obtained, with RSDr values lower than 7.5% in all cases, thus indicating the satisfactory suitability of the proposed methodology for the quantitative analysis of free and esterified HMC in different oil samples.

### 3.3. Application of the SPE-GC-FID Procedure to Real Samples

To check the applicability of the method and to obtain a range estimation of free and esterified HMC in pure EVOO, 15 EVOOs from different European countries (Spain, Italy, Greece, and Portugal) were collected and submitted to the in-house-validated SPE-GC-FID procedure. At the same time, 5 commercial refined sunflower oils were purchased at local market and analyzed. The results are reported in Table 2.

Although the number of EVOO samples analyzed was too limited to consider these data as universally acceptable, the results showed that free minor compounds in EVOO ranged from 1680 to 2390 mg/kg, while esterified ones were always lower than 1000 mg/kg, from a minimum of 360 to a maximum of 970 mg/kg, with a ratio between the two forms varying from 2.2 to 4.7. In sunflower oils, the range for free minor compounds was from 2310 to 3030 mg/kg and from 2660 to 2980 mg/kg for esterified minor components. Moreover, results confirmed high levels of Δ-7-stigmasteryl C18 in RSO, ranging from 800 to 910 mg/kg. In general, sunflower oils showed a very similar amount of esterified minor compounds and a wider range of free ones. These results confirmed the significantly higher level of esterified minor compounds in RSO compared to EVOO, thus revealing that the esterified fraction could represent the most diagnostic one for detecting the fraudulent addition of RSO to EVOO. Furthermore, it is interesting to highlight that the ratio between the two forms was always close to one and very similar in RSO (0.8–1.1) compared to the variability observed for EVOOs.

**Figure 2:**
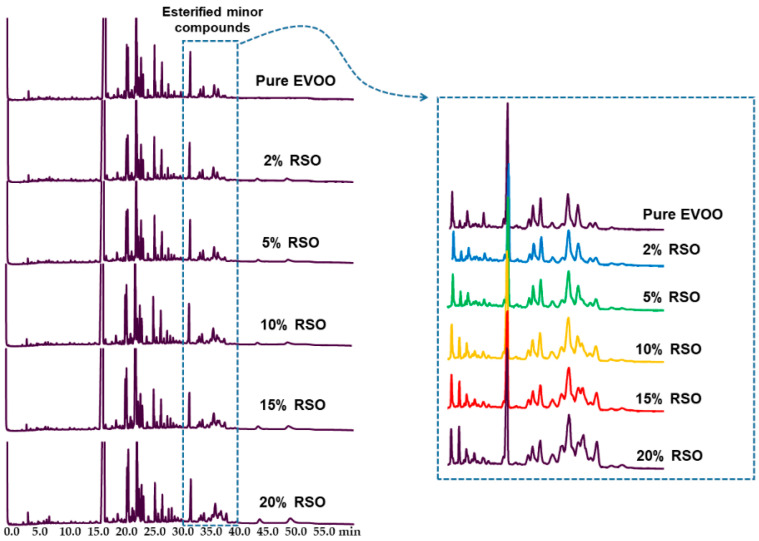
SPE-GC-FID chromatograms of TMS free and esterified HMC profiles of pure EVOO containing different percentages of RSO (2–20%). The expansion of the esterified minor compounds peak group is shown in the box.

Next, to determine the applicability of the methodology for quantification of HMC in illegal EVOO blends, a pure EVOO was selected and mixed with different amounts of RSO. For the study, several mixtures were prepared at different percentages equal to 2, 5, 10, 15, and 20% (*w*/*w*). In Figure 2, the GC traces obtained from analyzing the EVOO and RSO mixtures are presented. In the marked region, it can be seen that the levels of esterified minor compounds gradually increased when increasing the percentages of RSO in the blend.

In fact, as reported in Table 3, the method was able to provide a precise quantification of esterified minor compounds (theoretical—determined by considering the content in the oils composing the blends, as well as their presence in it—vs. experimental values), and to therefore detect even minor changes occurring in the esterified fraction content, as well as in the ratio between free and esterified forms upon the illegal addition of RSO to EVOO.

The small addition of 2% of RSO in EVOO led to an increase of the esterified minor compounds from 360 to 410 mg/kg, with a simultaneous decrease of free and esterified components ratio from 4.7 to 4.1. These trends were also confirmed for blends involving higher content of extraneous RSO.

**Figure 3:**
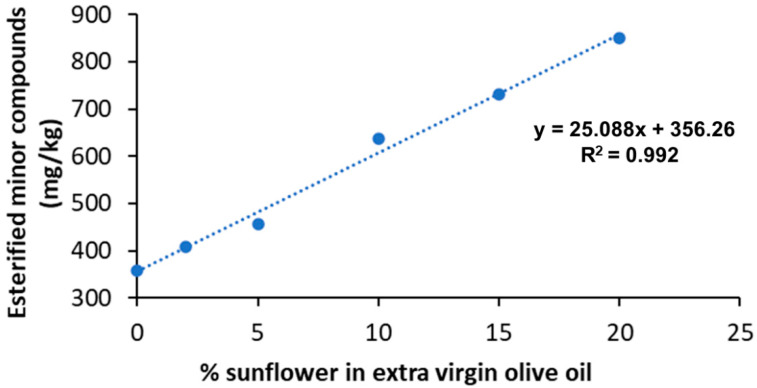
Linearity of esterified minor compounds concentrations in a pure EVOO and its blends with RSO at percentages of 2, 5, 10, 15, and 20% (*w*/*w*).

In fact, the levels of esterified compounds changed according to the amount of extraneous RSO added to the pure EVOO sample. Good linearity was achieved for EVOO–RSO blends, with correlation coefficients greater than 0.992 (Figure 3).

This capability can be of great interest for a future investigation into the establishment of possible parameters or limits that would be useful to detect the fraudulent addition of foreign refined oils in EVOO.

## 4. Conclusions

Olive oil is recognized as one of the food products that is at high risk of noncompliance and fraud. Therefore, great efforts are being made to develop an innovative analytical approach, as well as to improve already-existing official and nonofficial methods, for the detection of fraud in the olive oil sector. The method proposed in this study allows for reliable quantification of both free and esterified HMC in a single chromatographic run with the advantages, compared to the methodologies already proposed in the literature, of minimizing sample manipulation, volume of solvents and reagents, and time needed for the procedure. In addition, the proposed offline SPE-GC-FID methodology requires the use of simple instrumentation, which represents another crucial and positive aspect compared to other analytical approaches to assess olive oil purity based on more complex and expensive techniques (i.e., mass spectrometry, online LC-GC, etc.) that require highly expert operators. A reduction in toxicity of the solvents used also was obtained by substituting *n*-hexane with less-toxic isooctane, thus respecting the recent trends that are moving toward revision of existing methods with the aim to decrease the use of dangerous solvents in the routine laboratory.

The method was in-house validated, and the results showed good repeatability of the method carried out on pure EVOO, RO, and PO samples. Furthermore, the proposed methodology was also applied to EVOO mixed with different percentages of an extraneous oil (RSO), thus confirming its ability to detect changes of minor esterified compound levels in illegal blends.

In conclusion, the proposed quantification of free and esterified hydroxylated minor compounds might represent an opportunity to detect possible illegal oil admixtures containing seed oil, particularly RSO. Further studies, including large and exhaustive sampling procedures for the collection of pure olive and seed oils, are needed to establish limit ranges of free and esterified minor compounds and to assess the possible use of specific compounds as markers of fraudulent addition of extraneous seeds oils in olive oil.

## Figures and Tables

**Table 1:** Repeatability values for the optimized SPE-GC-FID method.

Sample	Free Minor Compounds * (mg/kg)	RSDr (%)	Esterified Minor Compounds ^₸^ (mg/kg)	RSDr (%)
EVOO	1840 ± 110	5.9	820 ± 40	4.6
PO	2130 ± 120	5.9	1400 ± 100	7.2
RSO	2400 ± 100	4.2	2460 ± 180	7.5

Results are expressed as average of free and esterified HMC content (mg/kg) of 6 replicates ± repeatability standard deviation. RSDr = repeatability relative standard deviation. * Sum of cholesterol, brassicasterol, campesterol, stigmasterol, Δ-7-stigmastenol, clerosterol, β-sitosterol, Δ-5-avenasterol, cycloartenol, 24-methylencycloartenol, Δ-5-23-stigmastadienol, sitostanol, Δ-5-24-stigmastadienol, and citrostadienol. ^₸^ Sum of campesteryl C18:1, sitosteryl C18:1, cicloartenyl C18:1, Δ-7-stigmasteryl C18, Δ-7-avenasteryl C18, 24 methylen cycloartenyl C18:1, and citrostadienyl C18:1.

**Table 2:** Free and esterified HMC content (mg/kg) and ratio in selected pure EVOO and RSO samples.

	Minor Compounds				
Sample		Free * (mg/kg)	Esterified ^₸^ (mg/kg)	Total (mg/kg)	Free/Esterified Ratio
Extra virgin olive oils	1a-SP	1970	770	2740	2.6
	2a-SP	1860	460	2320	4.0
	3a-GR	2390	970	3360	2.5
	4a-GR	2090	460	2550	4.5
	5a-GR	1680	360	2040	4.7
	6a-IT	2000	470	2470	4.3
	7a-GR	2040	470	2510	4.3
	8a-GR	2030	560	2590	4.0
	9a-GR	1940	490	2430	3.9
	10a-GR	1980	780	2760	2.5
	11a-GR	1920	490	2410	3.9
	12a-SP	2320	770	3090	3.0
	13a-IT	1790	460	2250	3.9
	14a-PR	1980	900	2880	2.2
	15a-PR	2210	770	2980	2.9
Sunflower oils	1b	2320	2980	5300	0.8
	2b	3030	2800	5830	1.1
	3b	2340	2730	5070	0.9
	4b	2530	2700	5230	0.9
	5b	2310	2660	4970	0.9

Results are expressed as average of free and esterified HMC content (mg/kg) of 3 replicates. Country of origin for EVOO samples: SP = Spain; GR = Greece; IT = Italy; PR = Portugal. * Sum of cholesterol, brassicasterol, campesterol, stigmasterol, Δ-7-stigmastenol, clerosterol, β-sitosterol, Δ-5-avenasterol, cycloartenol, 24-methylencycloartenol, Δ-5-23-stigmastadienol, sitostanol, Δ-5-24-stigmastadienol, and citrostadienol. ^₸^ Sum of campesteryl C18:1, sitosteryl C18:1, cicloartenyl C18:1, Δ-7-stigmasteryl C18, Δ-7-avenasteryl C18, 24 methylen cycloartenyl C18:1, and citrostadienyl C18:1.

**Table 3:** Content of free and esterified HMC (mg/kg) and ratio in pure EVOO and EVOO containing different percentages of RSO (2–20%).

		Free Minor Compounds *		Esterified Minor Compounds ^₸^		Free/Esterified Ratio
		Theoretical Level (mg/kg)	Experimental Level (mg/kg)	Theoretical Level (mg/kg)	Experimental Level (mg/kg)	
	EVOO		1680		360	4.7
%SO in EVOO	2	1690	1690	410	410	4.1
	5	1710	1760	480	460	3.8
	10	1740	1750	600	630	2.7
	15	1780	1740	720	730	2.4
	20	1820	1730	840	850	2.0

Results are expressed as average of free and esterified HMC content (mg/Kg) of 3 replicates. * Sum of cholesterol, brassicasterol, campesterol, stigmasterol, Δ-7-stigmastenol, clerosterol, β-sitosterol, Δ-5-avenasterol, cycloartenol, 24-methylencycloartenol, Δ-5-23-stigmastadienol, sitostanol, Δ-5-24-stigmastadienol, and citrostadienol. ^₸^ Sum of campesteryl C18:1, sitosteryl C18:1, cicloartenyl C18:1, Δ-7-stigmasteryl C18, Δ-7-avenasteryl C18, 24 methylen cycloartenyl C18:1, and citrostadienyl C18:1.

## Data Availability

The datasets generated for this study stored in an on-line repository are available on request to the corresponding author.

## Supplementary Materials

The following are available online at https://www.mdpi.com/article/10.3390/foods10061260/s1, Table S1: Relative retention time of compounds detected in samples employed for in-house validation.
