# Supplementary material for: In-House Validation of an SPE-GC-FID Method for the Detection of Free and Esterified Hydroxylated Minor Compounds in Virgin Olive Oils
Source: Foods. 2021 Jun 2;10(6):1260. doi: 10.3390/foods10061260 (PMC8230319; doi:10.3390/foods10061260)
Supplement: Supplementary file 1 [file foods-10-01260-s001.zip › foods-1198394-supplementary.pdf]

Table S1. Relative retention time of compounds detected in samples employed for in-house validation

**Peak identification**	
Free minor compounds	Relative retention time
Campesterol	1.05
Stigmasterol	1.06
Clerosterol	1.08
B-Sitosterol	1.10
Δ5 Avenasterol	1.10
Cicloartenol + Δ7 Stigmastenol	1.11
24-Methylencycloartenol	1.13
Citrostadienol + Erythrodiol	1.15
Esterified minor compounds	Relative retention time
Δ 7 Campesteryl C18:1	1.10
Sitosteryl C18:1	1.11
Cicloartenyl C18:1	1.12
Δ 7 Stigmasteryl C18	1.14
Δ 7 Avenasteryl C18	1.14
24 Methylen Cycloartenyl C18:1	1.15
Citrostadienyl C18:1	1.16
